# A comparison of self-report, systematic observation and third-party judgments of church attendance in a rural Fijian Village

**DOI:** 10.1371/journal.pone.0257160

**Published:** 2021-10-06

**Authors:** John H. Shaver, Thomas A. J. White, Patrick Vakaoti, Martin Lang

**Affiliations:** 1 Religion Programme, School of Social Sciences, University of Otago, Otago, New Zealand; 2 Centre for Research on Evolution, Belief and Behaviour, University of Otago, Otago, New Zealand; 3 Sociology Programme, School of Social Sciences, University of Otago, Otago, New Zealand; 4 Laboratory for the Experimental Research of Religion (LEVYNA), Masaryk University, Brno, Czechia; Coventry University, UNITED KINGDOM

## Abstract

Social desirability reporting leads to over estimations of church attendance. To date, researchers have treated over-reporting of church attendance as a general phenomenon, and have been unable to determine the demographic correlates of inaccuracy in these self-reports. By comparing over eight months of observational data on church attendance (n = 48 services) to self-report in a rural Fijian village, we find that 1) self-report does not reliably predict observed attendance, 2) women with two or more children (≥ 2) are more likely to over-report their attendance than women with fewer children (≤ 1), and 3) self-report of religiosity more reliably predicts observed church attendance than does self-report of church attendance. Further, we find that third-party judgements of church attendance by fellow villagers are more reliably associated with observed church attendance than self-report. Our findings suggest that researchers interested in estimating behavioral variation, particularly in domains susceptible to social desirability effects, should consider developing and employing third-party methods to mitigate biases inherent to self-report.

## Introduction

Social scientists continue to rely on self-reports for measuring behavior despite ongoing criticism concerning informant inaccuracy [1, 2]. Across various domains of human behavior, research finds that the unreliability of self-reports is due to individuals’ memory failure over time, including accidental omission and telescoping [3, 4], and cultural biases, including semantic similarity, prosocial signalling and identity-affirmation [1, 5, 6]. When reporting behavior of social and cultural significance, informants intuitively mishear value-laden questions [7], self-deceive and pursue impression management [8, 9].

Prominent in studies of informant inaccuracy, Socially Desirable Reporting (SDR) bias names the tendency of informants to over-report in ways that would be viewed as “good” by in-group members, and under-report “bad” behaviors [10, 11]. When informants inaccurately respond and instead report socially desirable behaviors it may be that, a) the informant believes what they report is accurate, despite its inaccuracy, or it may comprise b) an attempt at impression management, where the informant consciously presents themselves in a better light based upon perceived researcher expectations [9]. Regardless, SDR biases occur most often in response to questions about highly normative issues, such as childcare [12], abortion [13], diet-intake [14, 15], attitudes to asylum seekers [16], HIV status and drug use [17], domestic violence [18], sexual practices [19], voting for candidates from minority backgrounds [20], and religious participation, most notably church attendance [21].

The reliance on self-reports of church attendance as evidence for the resilience of church-going in the United States received a devastating critique in a 1993 study which showed data from self-reports exceeded that from direct observation by nearly two to one [21]. The study compared telephone self-reports of church attendance in Ashtabula County, Ohio, against headcounts conducted by the individual churches, along with estimates drawn from ‘the number of cars in [church] parking lots’ and ‘actually counting persons attending Sunday services’ for churches that did not return their own figures [21 p.744]. Interpreting over-reporting as due to SDR, the researchers offered that ‘if survey respondents view regular church attendance as normative or view infrequent church attendance as deviant, they may be inclined to overreport their attendance’[21 p. 749].

Subsequent efforts to improve the accuracy of self-reports of church attendance have focused on avoiding leading respondents towards over-reporting by asking about the topic indirectly. For example, asking informants on Mondays about what they did ‘yesterday’ avoids explicitly raising the issue of religion and church attendance [7]. Alternatively ‘time diaries’ may be completed in real time with participants completing entries at regular intervals throughout a given period, which may vary from a day to a much longer duration [22]. The indirect approach of time diaries successfully reduces self-reported attendance figures closer to those directly observed in counting methods.

Brenner advocates time-diaries to avoid identity-based responses where informants over-report their church attendance to convey more sincerely their religious identity [23, 24]. Moreover, Brenner, demonstrates how the discrepancy between accurate time-diaries and inaccurate conventional self-reports can reveal the relative importance of religious identity norms across cultures [5, 25]. Brenner argues that while higher rates of church attendance over-reporting in the United States compared to that in Europe might undermine claims of exceptional American religiosity by participation, it confirms claims of exceptional American religiosity by identity norms [5]. Alternative methods such as time diaries may not only improve data precision regarding measuring church attendance, they can show how different groups vary in their susceptibility to religious over-reporting. We suggest that where comparative data reveals such variation across other demographic variables, such as gender, age or child number, further inferences can be made concerning how norms of religiosity and religious behavior are experienced differently within a cultural group.

However, time diaries are not always ideal or practical for modelling biased reporting. Their disadvantages include: ‘lower subject compliance, missed occasions, undependable completion times, and a variety of response errors, many stemming from the cognitive demands of following an unfamiliar form and the burden of the initiative placed on the respondent’ [26 p.109]. Promptly completed time-diaries may mitigate inaccuracies from memory failure, but attaining their adequate completion can be difficult, particularly in cultural contexts where time-use is not rigorously regulated. Time diaries, moreover, are not immune to impression management bias, despite being indirect. For instance, ‘very few respondents report engaging in sexual or other biological activity in their diary accounts’ [27 p. 53].

In addition, time diaries *and* headcounts that measure church attendance typically involve asynchronous data-sets and sample different (but comparable) groups and individuals. While this is less problematic for estimating aggregate church populations across a geographical area, such imprecise data sampling is unhelpful for analysing demographic variation in over-reporting. One study supplementing time diary analysis with imputed data found that ‘no demographic covariants [sex, age, income, education, marital status or child number] are consistently associated with over-reporting’ [23 p. 111]. However, drawing on imputed data from different samples or using anonymous headcount data does not directly compare self-reports to actual attendance among the same individuals.

To the best of our knowledge researchers have yet to systematically and directly observe naturally occurring attendance at the level of individuals and compare this against their self-reports. These data are necessary for understanding the sources of self-report biases in church attendance. Below we compare the accuracy of self-reports to observed church attendance in a rural Fijian village over the course of 8+ months to uncover the demographic correlates of over-reporting.

Further, we compare these findings to the accuracy of third party judgements on church attendance, to evaluate these measures as an alternative to improve accuracy in estimations of behavioral variation. Third-party reporting on social behavior remains surprisingly under-utilised, and may offer an alternative method to self-report and time diaries. Typically third-party methods are employed in instances where the accuracy of informant self-reports are expected to be fallible, such as with cases of child abuse [28] or with adults with intellectual difficulties [29]. While less effective across large territories, in the small face-to-face communities where many social scientists work, third-party judgements may be useful as they do not suffer the biases of self-report, and may more accurately predict intracultural variation in highly prescribed social behavior, such as church attendance, which is monitored by many individuals in the group.

## Methods

### Field site

Data collection took place in a rural village on the island of Vanua Levu in Fiji’s Northern Division. The village sits on Savusavu Bay, approximately 48 km from the closest town of Savusavu, which has a population of 6,835 according to the last known census in 2017. The village is accessible via a 2.5 h bus ride and a 3 km hike from the bus stop to the village itself. When fieldwork took place (2009–2011) the population of the village varied but hovered around 90 people, with about 50 adults, 28 of whom were males. The village economy is a mix of subsistence production and wage earning from copra cutting. Men practice horticulture and cut copra while women fish and forage in Savusavu Bay.

Fiji’s religious diversity includes many Indo-Fijian Hindus and Muslims, but the Indigenous Fijian majority is near-exclusively Christian, and Fiji’s colonial legacy of Indigenous protectionism and racial segregation left most villages singularly Christian. Fijian Christianity is dominated by the Methodism preached by European missionaries who arrived to the islands in 1835. Fijians integrated the missionaries’ Methodism with the traditional religious system centred on the *vanua*, an Indigenous holistic category comprising the land, the ancestors, the people and wildlife of the area. Fiji’s Methodist church (*lotu*) has remained strongest in rural areas, where an alliance between church ministers (*talatala*) and Fiji’s chiefs (*turaga*) has better withstood competition from later arriving Pentecostal and Adventist churches. Today the main rituals of the *lotu* are church services. Such services are frequent and are the center of village activities, especially on Sundays. In the village where this research took place, church services occur at least three times on Sundays, and sometimes on other days throughout the week. There are always three church services on a Sunday: at 7:00, 10:00, and 15:00. Every day where this research took place, villagers engage in daily village-wide prayer four times (at 6:00am, 12:00pm, 6:00pm, and 12:00am). Upon the beating of a traditional wooden drum (*loli*) villagers are expected to stop what they are doing and engage in prayer.

### Participants

We attempted to recruit all resident adult members of the village in all rounds of data collection. Out of the 50 regular village residents, 45 (25 males, 20 females, with ages ranging from 20–78 and a mean age of 46.72) completed a short survey that asked about religious beliefs and behavior. On the day informants completed these surveys, there were 47 adults in the village, so these data represent 96 percent of the population of the village at that time. The two villagers (a husband and wife) present in the village in the time of survey data collection refused to answer the survey because they previously conversed on similar topics with JS. Study protocols were approved by the University of Connecticut Institutional Review Board and informants provided written consent for household censuses, and the self-report and third-party judgement surveys. All data and analysis scripts are publicly available on the Open Science Framework (https://osf.io/7vp9m/).

### Household censuses

A census of all village households (n = 20) took place from December, 2009 –March, 2010. The census included collecting demographic (household residents, age, sex, marital status) and kinship data. At the time of data collection, there was no secondary school in the area of the village. When children reached the age of 14, they left the village to reside with relatives who lived close to a secondary school. As a result, there were no children between 14–18 in the village during fieldwork.

### Self-report surveys

In addition to questions regarding local ancestor beliefs (see [30]), nine questions aimed at measuring commitment to the Christian belief system were drawn from a modified form of a cross-culturally tested religiosity scale [31–33]. These questions related to participants’ beliefs (e.g., god’s existence), church attendance and practices (e.g., bible study), and experiences (e.g., feeling of god’s presence) and were answered on a 5-point scale. The full survey can be found in the S1 File (items were reversed for the analysis). Out of these nine questions, we singled out self-reported church attendance (from “Every time” to “Never”) as our main predictor variable of observed attendance (see below).

Surveys were translated from English to Fijian and then back-translated to English to check for accuracy. Informants read and filled out the surveys themselves at the village community hall. Surveys were completed during the first week of April, 2010.

A variable by variable analysis revealed that responses to some of the nine variables exhibited extremely low variance. For example, for the question on belief in God, 37 participants chose the highest score on the scale (“I am sure that God exists and is active in my life”) while 8 participants answered one categorical response lower (“Although I sometimes question God’s existence, I do believe in God, and believe that he knows of me as a person”), leaving most of the 5-point scale unused. Further items with low variation included questions about daily practice of prayer and a question about the degree to which Christianity gives comfort and security. While disqualifying these items from further analysis (see S1 File for factor loadings of these items), the low variance and ceiling scores on these items illustrate high levels of belief in the Christian God across the studied population. The latent variable related to commitment to Christian beliefs was therefore constructed out of 5 variables that exhibited sufficient factor loadings and an adequate Cronbach’s alpha (α = 0.69). See S1 File for details on the factor analysis.

### Observed church attendance

Once every adult member of the village was known by sight, observational data collection of church attendance began. Observations occurred across 48 church services from April 28th, 2010 –December 5^th^, 2010. The church minister, the village church elder, and the hereditary chief *(turaga ni yavusa)* were all informed about the wish to collect data on church attendance, and all consented. These three men, along with a research assistant, were the only people notified about the project. These individuals were asked not to inform other village members of the data collection, in the hopes of not altering naturally occurring attendance in a public space.

To record church attendance, a research assistant and JS sat in the back of the church and repeatedly scanned all of the adult attendees throughout each service. Both JS and the research assistant attended all services included in subsequent analyses. The research assistant was also the elected *(turaga ni koro)*, an executive office–separate to hereditary chieftainship–with responsibility for ensuring compliance with village by-laws. Compliance with this local customary law required individuals to notify the *turaga ni koro* when they left the village, say where they were headed and also declare when they returned. Thus if an individual did not attend the church service and was not in the village, with consultation of the *turaga ni koro*, it was possible to note his or her location. To avoid disruption, and to maintain proper decorum during church services, attendance registers were compiled immediately after the service at JS’s residence.

Once back at JS’s residence, JS and the *turaga ni koro* separately filled out previously prepared sheets and recorded whether or not a person attended, and if they did not attend if they were present in the village. In all recordings there were no discrepancies between the observed attendances noted by JS and the *turaga ni koro*. Those in the village were coded “at risk” of attendance. These methods resulted in observational data on a total of 50 individuals. The mode of events at risk was 48 while the mean was 42 and minimum 24. Of the 48 services observed, the majority took place on Sundays (n = 41), with others less frequently observed throughout the week (Mondays: n = 1; Tuesdays: n = 1; Wednesdays: n = 3; Thursdays: n = 1; Saturdays: n = 1). As part of Fiji’s ongoing tradition of Sabbatarianism, Sunday attendance is prioritised in the village with weekday attendance broadly viewed as additional and not substitutional to Sunday worship.

### Third party judgements

On November 15^th^, 2010, informants completed anonymous surveys that asked them to list other village members who embody several traits or most frequently engage in a specific behavior both positive and negative, including the most hardworking, the most cooperative, the best fishers, the most frequent kava drinkers (typically seen as a trait associated with infrequent church attendance, see [34]) and the most frequent churchgoers. Informants were asked to list the top five men and top five women who most embodied the trait, or most frequently engaged in that behavior. On the morning of data collection, surveys and pencils were passed out to everyone in the village at their homes. After completing the surveys, informants returned the surveys by placing them in a large manila envelope at J.S.’s residence where they were paid $2 FJ.

Combined men and women completed 46 surveys. Surveys asked informants to indicate their sex, but very few actually did. All surveys were anonymous so this flaw in the survey instrument was not known until the completion of all surveys. As a result of this error, the data from all surveys are analyzed together and no possible sex differences can be examined. However, given the small closed nature of this group and the public nature of the behaviors measured in these surveys, it is unlikely that men and women differ significantly in their rankings. Each person was assigned a score of 5 if s/he was ranked first, a 4 if s/he was ranked second, and so on down to 1 for being ranked fifth. These scores for each individual were then summed across surveys for each individual, for each dimension of reputation. This resulted in reputation scores that varied from 0 to a possible 230 (a person listed first across 46 surveys).

### Analysis

All data were analysed in R, version 3.6.3 [35]. To examine the accuracy of self- reports, our main outcome variable was observed attendance at Christian services for each individual with attendance at 48 possible events. Not all participants were present in the village for all 48 events, thus we excluded events for individual participants when they were not at risk (i.e., participants had a variable number of events they could attend). To account for the uneven number of events at risk between participants, we model the probability of participation at each event that participants were at risk rather than summing the number of attended church services for each individual, which would give biased estimates due to the uneven number of events at risk. That is, our basic unit of analysis is an event for an individual, together comprising 2101 data points (on average 42 events per participant). To account for the fact that attendance at the 48 events is correlated for individual participants, we let the intercept vary for each participant and the models include participant-level (rather than event-level) predictors. Specifically, we modelled the probability of attendance with generalised linear mixed models (GLMMs) using the *glmer* command from the *lme4* package [36] and setting the family as “binomial” (with logit link). The general structure of our models is as follows:

$$g(Y_{i})=((\beta_{i0}+u_{0j})+X_{j1..k}\beta_{j1\ldots k})∼Binomial(n,p)$$

where *Y*_*i*_ is the probability of attendance at an event transformed with the logit link *g*. *β*_*i0*_ is a fixed intercept for an event and *u0j* is a varying intercept for individual participants. *X*_*j1*..*k*_ are participant-level predictors (self-reported attendance, religiosity, third-party judgment, sex, age, number of children) and *β*_*j1…k*_ estimated regression coefficients for each predictor. Note that we decided to analyse the data at the lowest possible level (participant-event risk) so as not to lose information by transforming the outcome variable. However, transforming the outcome variable to the percentage of at risk events attended and using a generalised linear model with a beta family to model this percentage yields highly similar results (see https://osf.io/7vp9m/ for details).

Five participants did not provide data on religiosity and one of those participants did not know their year of birth. Since these missing data are not conditional on the outcome variable (see S1 File), complete-case analysis should be comparable to multiple imputation [37]. Thus, in the main text, we report the complete-case analysis but display models with imputed data in the S1 File, section S1.2 (and see this section for further discussion of multiple imputation).

Though we sampled nearly the whole population, our sample size is rather small, and thus we built parsimonious models including only the main predictor of interest or an interaction between two predictors. An exception to this are models where we aimed to compare the relative importance of variables in predicting the probability of attendance. Relative predictor importance was estimated with the semi-partial R^2^ statistic (R^2^_β*_) developed by Jaeger et al. [38] for GLMMs, using the command *r2beta* from the package *r2glmm*. The semi-partial R^2^_β*_ expresses the association between the outcome and single predictor variable while accounting for other predictors in the model. As a general rule, we opted to first display the estimated relationship between the outcome variable and its predictors when individually added to the models, and then compared the strength of those predictors within one model using standardized coefficients and the variance explained using the R^2^_β*_ statistic. See S1 File, section S1.3 for a discussion of potential caveats that should be considered with this method.

## Results

### Predictors of observed attendance

**Fig 1:**
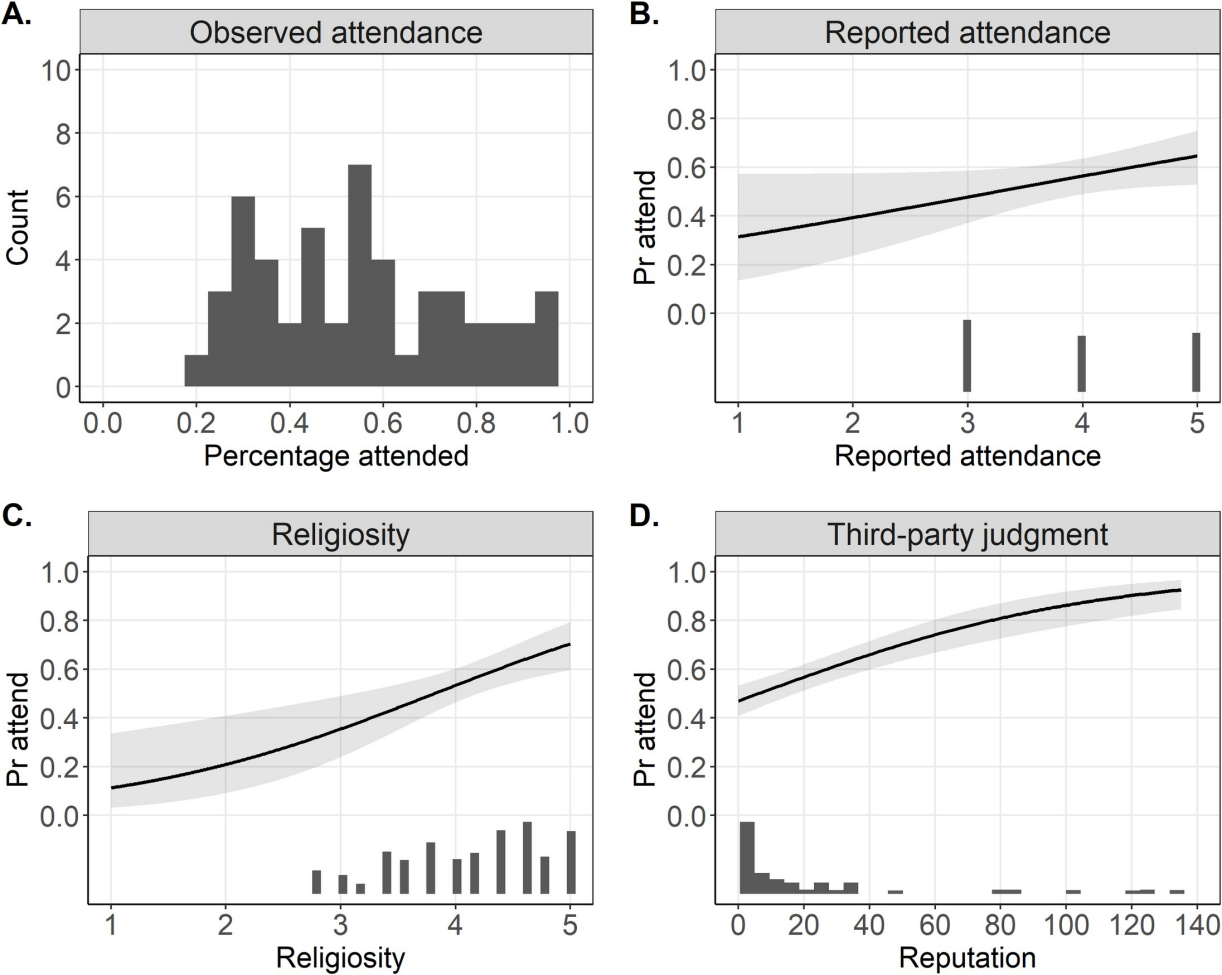
Observed attendance and its predictors. A–D report logit regression estimates with 95% CIs and underlying histograms of predictors. **A.** Histogram of percentage of attended events when at risk. **B.** Self-reported attendance was only weakly associated with observed attendance, partially due to the low variability of this scale. **C.** Likewise, religiosity revealed low variability, albeit its association with observed attendance was stronger than self-reported attendance. **D.** Third-party judgements of attendance was the strongest predictor of observed attendance, as supported by the semi-partial R^2^ measure (R^2^_β*j_).

Informants attended just over half (55%) of all possible events (2101), suggesting that the odds of attending were 1.31 for an average individual. The participant with the lowest attendance took part in 21% of all events when at risk, while the most frequent attendee took part in 96% of possible events. As a first predictor of observed attendance, we used the self-reported attendance (Never–Rarely–Sometimes–Frequently–Everytime) to check whether self-reported attendance is a reliable predictor of observed attendance. However, as displayed in Fig 1A, self-reported attendance displayed relatively low variability (M_self-reported_ = 3.91, SD = 0.85; see Fig 1B for histogram), similar to other religiosity measures. While participants were distributed rather equally across the three highest options, no one reported that they “rarely” or “never” attend church. This is consistent with ethnographic observations, which suggest that there was no one in the village who never attended. Related to this shortcoming, self-reported attendance was only weakly correlated with the probability of observed attendance (OR = 1.41; 95% CIs = [0.99, 2.04]). Specifically, scoring “sometimes” on the self-reported scale was associated with 48% probability of attending a single event while scoring “everytime” was associated with a 65% probability of attendance.

The next variable associated with observed attendance we considered was self-reported commitment to the Christian belief system (i.e., religiosity). Since religiosity was constructed as a latent variable comprising five items (not including self-reported church attendance), we observed more nuanced inter-individual variation in this predictor compared to self-reported church attendance, albeit none of the participants scored on the lower portion of this variable (M_religiosity_ = 4.12, SD = 0.64; see Fig 1C for histogram). In the GLMM framework, moving one point on the religiosity score was associated with increasing the odds of observed attendance by 2.08 (95% CIs = [1.34, 3.27]). While the lowest scoring participant on this scale had the probability of attending a single event estimated at 32%, the highest scoring participant had a probability of 67%.

Finally, we also explored whether third-party judgements of church attendance obtained from a villager’s peers would be predictive of observed attendance. The sum of individual nominations (including the position in the nomination list) revealed that the mode of this measure was 0 (no nomination for 16 out of 50 villagers) and the maximum obtained nomination-points was 135 (M_repuatation_ = 20.28, SD = 35.08; see Fig 1D for histogram). The GLMM estimated that an increase in ten nomination-points was associated with a 10.20 higher odds of observed attendance and this effect was well estimated (95% CIs = [10.13, 10.27]). For the non-nominated, the probability of attending was estimated at 47%, and for the most nominated person, the probability of attendance at each event was estimated at 93%. See Fig 1 for a complete visual overview of these results.

We standardized these three predictors (reported attendance, religiosity and third-party judgements) such that they would have a M = 0 and SD = 1 and compared their relative strength in one model. Similar to the results reported above, third-party judgements of church attendance was the strongest predictor: a change of 1 SD on a nomination score was associated with an increased odds of attendance of 1.87, while for religiosity this increase was estimated at 1.17, and for self-reported attendance at 1.09 (Table 1). Together, these three variables were estimated to explain 17% (R^2^_β*_ = 0.169) of variance in the observed attendance variable. The semi-partial R^2^ for third-party judgements was estimated at R^2^_β*j_ = 0.101, for religiosity it was estimated at R^2^_β*j_ = 0.006 and for self-reported attendance it was estimated at R^2^_β*j_ = 0.002.

**Table 1:** Raw estimated with 95% CIs from GLMMs reporting the coefficients for self-reported, peer-reported, and demographic variables predicting observed attendance at church services.

	Observed attendance	
	(1)	(2)
Intercept	0.26	-0.62
	(0.04, 0.49)	(-1.95, 0.71)
Self-reported attendance	0.09	--
	(-0.16, 0.34)	
Religiosity	0.16	--
	(-0.12, 0.43)	
Third-party judgment	0.63	--
	(0.35, 0.90)	
Age	--	0.03
		(0.01, 0.05)
Sex	--	0.34
		(-0.21, 0.89)
Education	--	0.08
		(-0.06, 0.21)
N Observations	1,877	2,055
N Participants	45	49

Note. Self-reported attendance, religiosity, and reputation are z-scored. Age is centered at its mean.

### Demographic correlates of inaccuracy in self-report

**Fig 2:**
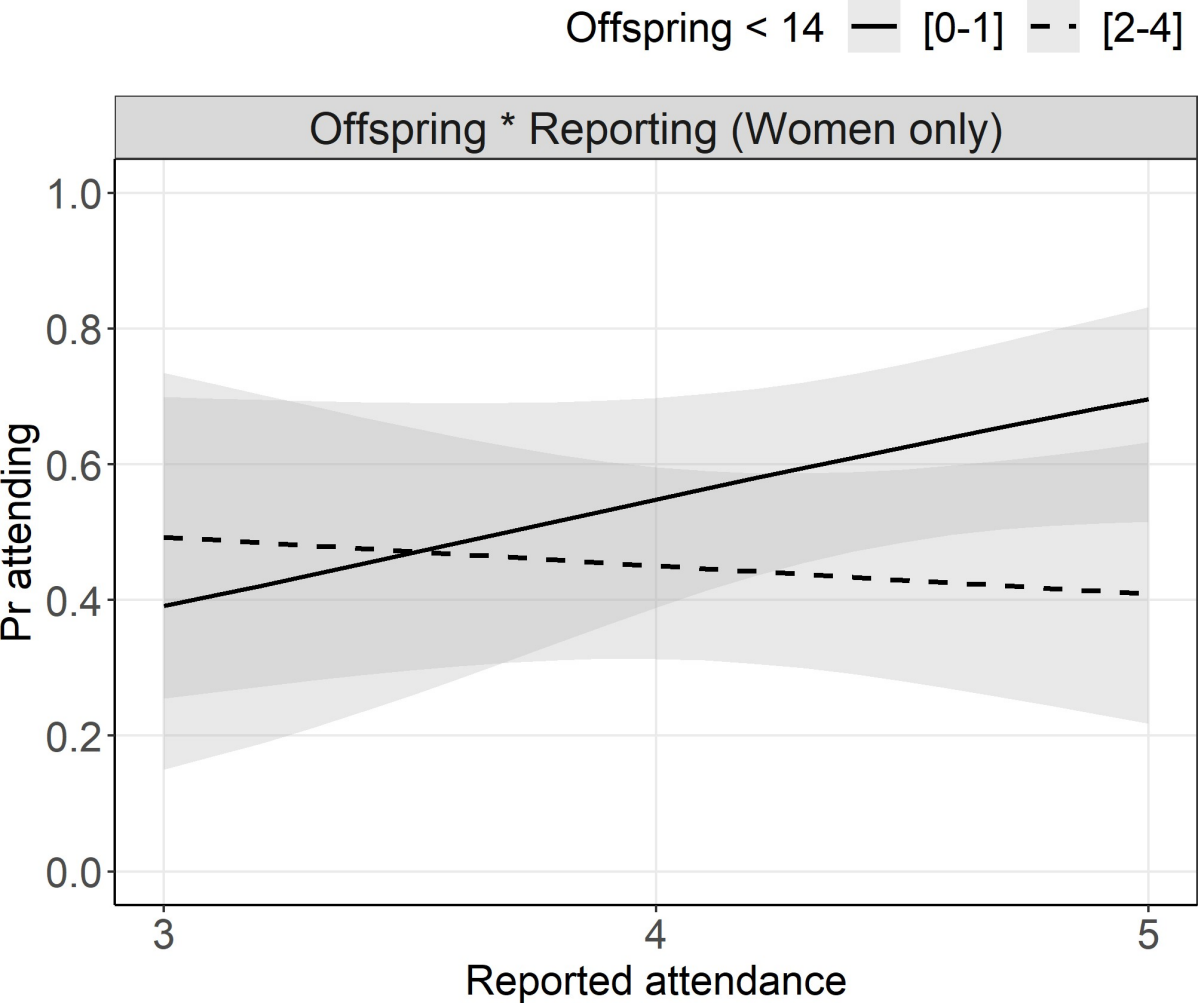
Observed attendance by reported attendance among women with different numbers of offspring. Regression lines with 95% CIs. Self-reported attendance was positively associated with observed attendance for women with zero to one child (n = 13), but this relationship was reversed for women with two or more children (n = 9). Note that this plot is a simplified illustration of the statistical model where the number of children was treated as a continuous variable.

As the self-reported measure of attendance revealed a relatively poor association with observed attendance, we explored further variables that could explain biases in self-reports. As a starting point, we estimated the effects of demographic variables on observed attendance to identify groups with lower attendance that may result in self-reporting bias. Investigating the effects of sex, age, and education on observed attendance, we found that older and less educated participants were more likely to attend, as were males (albeit these effects were precisely estimated only for age (Table 1)). Ethnographic observations conducted by JS suggested that (especially younger) women are burdened with childcare, which could decrease their chances of attendance and possibly lead to an over-reporting bias among these individuals. Indeed, modelling the probability of attendance for women using the interaction between the number of resident offspring under 14 years of age and self-reported attendance revealed that more resident offspring was associated with a greater discrepancy between self-reported and observed attendance. While the coefficient for self-reported attendance was positive for women with no resident offspring (OR = 2.42, 95% CIs = [1.05, 5.81]), this coefficient was estimated to weaken with each additional child by an odds of 0.58 (95% CIs = [0.34, 0.97]). The simplified results are displayed in Fig 2. for illustration. Note that due to the relatively small sample size, the confidence of the estimates is wide.

## Discussion

Our results suggest that self-report of church attendance in a rural Fijian village is inaccurate, but that some members within the village are more likely to over-report than others. Specifically, each additional child under 14 years was associated with higher rates of women over-estimating their attendance. Speculating, it could be that women with several children report their attendance levels prior to high childcare burdens, or that they simply report how much they *would* go if they were not to have several children. Alternatively, these women might be more susceptible to SDR due to often requiring allocare support from other village members, which is frequent in Fijian villages, settings in which having a reputation for being religious is associated with positive social standing and increases in received cooperation [34].Through its more precise sampling, our study’s analysis of variation in over-reporting by gender has been able to go beyond accounts that stop at the general higher religiosity of women (e.g., [24]). Moreover, our results suggest that the accuracy of self-report of church attendance is affected by the local economic division of labor, particularly with respect to norms for childcare. Economic divisions of labor vary cross-culturally, and it is likely that self-report biases in church attendance will vary according to cultural differences in childcare norms.

We find that self-reported measures of religiosity are better associated with observed church attendance than self-reported attendance. Finally, we find that third-party judgements of church attendance are more reliably associated with observed attendance than self-reported church attendance or religiosity. This finding is in line with research on the costly signalling theory of ritual behavior [39] and suggests that community members do pay close attention to ritual signals.

A potential rejoinder to the low accuracy of self-reports might focus on the form of questioning employed in this study. The imprecision of the ‘how often’ question style may have drawn respondents to offer a generally-assumed rate of occurrence rather than the actual frequency of their church attendance. Along with the undefined timeframe for respondents to base their responses, this may have helped villagers look past any recent absenteeism [40]. However, not only have Vezzoni and Biolcati challenged the extent to which the ‘how often’ question actually produces this bias, they argue that a more definite timeframe may just as likely undercount recently-absent, normally high attenders as an indefinite timeframe may overcount regular absentees [41].

We were able to compare self-reports to naturally occurring attendance because of the small face-to-face nature the study population, and we expect that these methods, and third-party measures more generally, could be fruitfully employed among populations where all group members are known to one another. Indeed, this research could have been conducted without issue in any Fijian village (though the village in the current study is on smaller side of Fijian villages), and in many settings where social scientists work. However, the methods we employed are not readily adaptable to large social groups where community members may be unknown to the researcher.

Methods other than self-report and third-party measures are likely more applicable in large populations where there is large variation in religious behavior, and particularly in settings where population level estimates of religious behavior are of interest. In a novel study, Gervais and Najle [42] used the unmatched count technique to estimate levels of atheism in the United States, where belief in God is prescribed and atheism is often stigmatized. The findings of Gervais and Najle suggest that rates of atheism in the United States are at least twice as high as estimates derived from self-report. These methods have been employed in a variety of domains where SDR is expected [42], and could be further expanded to determine population level estimates of religious belief and behavior in post-industrial settings.

In other words, the suitability of methods for assessing church attendance, religiosity, and/or supernatural belief levels vary according to the size of the population of interest. The appropriateness of a method also depends on the research questions of interest. Researchers employing self-report surveys or time diaries on weekly church attendance are often interested in changes in attendance over time, particularly as they may point to broad patterns of secularization. Third-party measures are unlikely to be as useful as self-report surveys for understanding changes in attendance over time, as they are difficult to scale-up to large populations, and the repeated use of third-party surveys is not often practical. Our results suggest that third-party measures may better capture within group variation in church attendance than self-report measures at a fixed time point. Our current third-party measure, however, does not provide a measure of attendance for any one individual, nor for a population-level estimate of attendance. Moreover, our data indicate that individuals living in Fijian villages are highly religious and it remains unknown whether third-party measures can capture variation in religious behavior in less religious settings.

The past twenty to thirty years has seen a tremendous increase in the scientific study of religion [33, 43], and many of these studies rely upon self-report. Our findings call into question the accuracy of some these data, at least in terms of how they operationalize variation in religious behavior, and suggest that the development of third-party judgments is warranted, particularly for use in smaller communities.

## Supporting information

S1 File (DOCX)
