# Supplementary material for: A comparison of self-report, systematic observation and third-party judgments of church attendance in a rural Fijian Village
Source: PLoS One. 2021 Oct 6;16(10):e0257160. doi: 10.1371/journal.pone.0257160 (PMC8494351; doi:10.1371/journal.pone.0257160)
Supplement: S1 File — (DOCX) [file pone.0257160.s001.docx]

**Supporting Information for A Comparison of Self-report, Systematic Observation and Third-party Judgments of Church Attendance in a Rural Fijian Village, by Shaver, White, Vakaoti and Lang.**

## Scales

We used factor analysis with oblique rotation (“oblimin”) to assess whether the variables representing commitment to the Christian belief system load on a single factor as expected. Bartlett’s test of sphericity indicated that all items were sufficiently inter-correlated (χ2 (28) = 3103, p < .001) but the Kaiser-Meyer-Olkin (KMO) test revealed that sampling was not adequate (MSA = .54) with prayer frequency, belief, and comfort variables having the lowest scores. This problem was also reflected in the factor loadings, which revealed that the belief variable had a negative loading on the latent commitment concept (loading = -0.19) and the prayer and comfort variables loaded only weakly (loadings 0.22 and 0.39, respectively). Including these three variables into the latent commitment concept yielded a relatively low Cronbach’s alpha value of 0.54. As the one-factor solution revealed three variables with low factor loadings, we also assessed a two-factor solution. However, the second factor would not help to retain the problematic variables in the analysis. While prayer frequency dominated this factor (loading = 0.88), the loading of belief on the second factor was -0.05 and -0.33 for  relig_comfort, supporting a one-factor solution. The final latent concept from the one-factor solution therefore comprised five variables and revealed a sufficient Cronbach’s alpha of 0.69. See Tab. S1 for factor loadings and eigenvalues.

| **Tab. S1**. Factor loadings of commitment to Christian belief (religiosity). | | |
| --- | --- | --- |
| Variable | Christian commitment | |
|  | (1) | (2) |
| Q1 | 0.22 | -- |
| Q2 | 0.66 | 0.67 |
| Q3 | 0.62 | 0.71 |
| Q4 | -0.19 | -- |
| Q5 | 0.66 | 0.66 |
| Q6 | 0.74 | 0.69 |
| Q7 | 0.39 | -- |
| Q9 | 0.58 | 0.62 |
| Eigenvalue | 2.38 | 2.24 |
| Cronbach’s α | 0.54 | 0.69 |
| Note. Question numbers reflect questions in section S1.4. | | |

## Models with imputed data

To impute the missing predictor data, we used the multivariate imputation by chain equations procedure in R [1]. Specifically, we used the command *mice* from the *mice* package, setting the number of simulated datasets at 50 as a compromise between capturing the full uncertainty of estimates and computing time. The *mice* command simulates 50 datasets by randomly imputing data from pre-defined distributions of values within the range of each variable and conditioned on other variables in the dataset. That is, we generated the imputed datasets using the predictive mean matching algorithm with the full dataset as predictors of the imputed values. Note that we used the composite latent variable scores for imputation rather than the individual scale items such that we could pool the model estimates in the next step of analysis. This procedure yielded 50 datasets that were used to test our hypotheses. To get at single estimates for each model that would reflect the uncertainty of the imputed data across the 50 datasets, we used Rubin’s rules to pool parameter estimates from the 50 models (using the *pool* command from the mice package). As such, we were able to fit the GLMM as in the main text and predict observed attendance with a larger sample size. The results are displayed in Table S2. They are qualitatively comparable to the complete-case analysis, suggesting that for our data sets, the former analysis is preferable.

| **Table S2.** Raw estimated with 95% cIs from GLMMs reporting the coefficients for self-reported, peer-reported, and demographic variables predicting observed attendance of church services. Estimates from imputed models. | | |
| --- | --- | --- |
|  | Observed attendance | |
|  | (1) | (2) |
| Intercept | 0.30 | -0.49 |
|  | (0.08, 0.51) | (-1.82, 0.84) |
| Self-reported attendance | 0.10 | -- |
|  | (-0.15, 0.34) |  |
| Religiosity | 0.14 | -- |
|  | (-0.12, 0.40) |  |
| Third-party judgment | 0.61 | -- |
|  | (0.36, 0.87) |  |
| Age | -- | 0.03 |
|  |  | (0.01, 0.05) |
| Sex | -- | 0.29 |
|  |  | (-0.25, 0.84) |
| Education | -- | 0.07 |
|  |  | (-0.07, 0.20) |
| N Observations | 2,101 | 2,101 |
| N Participants | 50 | 50 |
| Note. Self-reported attendance, religiosity, and reputation are z-scored. Age is centered at its mean. | | |

## Discussion of R^2^

While informative, the estimates of R^2^ have several caveats and estimating relative predictor importance is a subject to an ongoing debate even for simple linear regression models [2]. Currently, there is no consensus on the best approach of extending these metrics to LMMs [3–5] and, subsequently, GLMMs [6]. The approach taken in the current paper relies on general properties of R^2^ to provide the proportion of explained vs. unexplained variance without partitioning the explained variance between the fixed and random effects [6]. Jaeger et al. [7] compare the null and full model with identical covariance structures using the F-statistic for a Wald test to calculate a single measure of variance explained by fixed effects [3]. Using the penalized quasi-likelihood estimator that transforms GLMM into a pseudo LMM, Jaeger et al. [7] extend the approach developed by Edwards et al. [3] to calculate R^2^_β*_ where β∗ reflects the generalization of R^2^_β_. Note that the authors of the r2glmm package caution against using their measure for logistic regression with a low number of events, however, this should not be a problem with our analyses, and together with the strength of the coefficients, we are relatively confident with the use of this measure in our analysis.

References

1. Buuren S van, Groothuis-Oudshoorn K. mice: Multivariate imputation by chained equations in R. J Stat Softw. 2010; 1–68.

2. Grömping U. Variable importance in regression models. Wiley Interdiscip Rev Comput Stat. 2015;7: 137–152. doi:10.1002/wics.1346

3. Edwards LJ, Muller KE, Wolfinger RD, Qaqish BF, Schabenberger O. An R2 statistic for fixed effects in the linear mixed model. Stat Med. 2008;27: 6137–6157. doi:10.1002/sim

4. Snijders TAB, Bosker RJ. Modeled Variance in Two-Level Models. Sociol Methods Res. 1994;22: 342–363. doi:10.1177/0049124194022003004

5. LaHuis DM, Hartman MJ, Hakoyama S, Clark PC. Explained Variance Measures for Multilevel Models. Organ Res Methods. 2014;17: 433–451. doi:10.1177/1094428114541701

6. Nakagawa S, Schielzeth H. A general and simple method for obtaining R2 from generalized linear mixed-effects models. Methods Ecol Evol. 2013;4: 133–142. doi:10.1111/j.2041-210x.2012.00261.x

7. Jaeger BC, Edwards LJ, Das K, Sen PK. An R2 statistic for fixed effects in the generalized linear mixed model. J Appl Stat. 2017;44: 1086–1105. doi:10.1080/02664763.2016.1193725

## Survey

1. Which of the following BEST describes your practice of prayer?
   1. Prayer is a regular part of my daily life
   2. I only pray during times of stress and need
   3. I only pray during formal ceremonies and at meals
   4. Prayer is of little importance to me
   5. I never pray
2. If you have a serious personal problem, how often do you take religious teachings (when translated literally teaching of the church/Christianity) into consideration?
   1. Almost always
   2. Usually
   3. Sometimes
   4. Rarely
   5. Never
3. How much does Christianity influence how you choose to act and spend time each day?
   1. A large amount
   2. A fair amount
   3. Somewhat
   4. A small amount
   5. Not at all
4. Which of the following statements comes closest to your belief in God?
   1. I am sure that God exists and is active in my life
   2. Although I sometimes question god’s existence, I do believe in God, and believe that he knows of me as a person
   3. I don’t know if there is a personal God, but I believe there is a higher power of some kind
   4. I don’t know If there is a god or higher power of some kind and I don’t know if I will ever know if there is a God
   5. I do not believe in a personal god or a higher power
5. During the past year, how often have you experienced a feeling of religious reverence (When translated the present of the Christian God)?
   1. Daily
   2. Frequently
   3. Sometimes
   4. Rarely
   5. Never
6. How often do you study the bible?
   1. Daily
   2. At least once a week
   3. At least once a month
   4. A few times a year
   5. Never
7. How much do you agree with the following statement?

Religion gives me a strong amount of comfort and security in my life.

- 1. Strongly agree
  2. Agree
  3. Uncertain
  4. Disagree
  5. Strongly disagree

1. How often do you attend church?
   1. Everytime
   2. Most of the time
   3. Sometimes
   4. Rarely
   5. Never
2. Which of the following statements comes closest to your belief about life after death?
   1. I believe in a personal life after death, a soul existing as a specific individual
   2. I believe in a soul existing after death as part of a universal spirit
   3. I belief in life after death of some kind, but I don’t really know what it would be like
   4. I don’t know whether there is any kind of life after death, and I don’t know if I will ever know
   5. I don’t believe in any kind of life after death
3. How often do you attend church meetings?
   1. Everytime
   2. Most of the time
   3. Sometimes
   4. Rarely
   5. Never
4. Are you?
   1. Baptized
   2. Committed Christian
5. Are you a member of the church choir?
   1. Yes
   2. No
6. Are you certified to preach in church?
   1. Yes
   2. No
